# Supplementary material for: Atomic origin of spin-valve magnetoresistance at the SrRuO3 grain boundary
Source: Natl Sci Rev. 2020 Jan 21;7(4):755–62. doi: 10.1093/nsr/nwaa004 (PMC8288863; doi:10.1093/nsr/nwaa004)
Supplement: nwaa004_Supplemental_File [file nwaa004_supplemental_file.docx]

Supplementary Information for

**Atomic Origin of Spin-Valve Magnetoresistance at the SrRuO_3_ Grain Boundary**

Xujing Li^1,2,‡^, Li Yin^3,‡^, Zhengxun Lai^3^, Mei Wu^1,4^, Yu Sheng^5^, Lei Zhang^2^, Yuanwei Sun^1,4^, Shulin Chen^1^, Xiaomei Li^2^, Jingmin Zhang^1^, Yuehui Li^1,4^, Kaihui Liu^6,7^, Kaiyou Wang^5,8^, Dapeng Yu^1,6,9^, Xuedong Bai^2,7,^*, Wenbo Mi^3,^*, Peng Gao^1,4,7,^*

^1^Electron Microscopy Laboratory, School of Physics, Peking University, Beijing, 100871, China

^2^Beijing National Laboratory for Condensed Matter Physics and Institute of Physics, Chinese Academy of Sciences, Beijing 100190, China

^3^Tianjin Key Laboratory of Low Dimensional Materials Physics and Preparation Technology, School of Science, Tianjin University, Tianjin 300354, China

^4^International Center for Quantum Materials, School of Physics, Peking University, Beijing, 100871, China

^5^State Key Laboratory of Superlattices and Microstructures, Institute of Semiconductors, Chinese Academy of Sciences, Beijing 100083, China

^6^ State Key Laboratory for Artificial Microstructure & Mesoscopic Physics, School of Physics, Peking University, Beijing 100871, China

^7^Collaborative Innovation Centre of Quantum Matter, Beijing 100871, China

^8^ College of Materials science and Opto-Electronic Technology, University of Chinese Academy of Sciences, Beijing 100049, P. R. China

^9^Shenzhen Institute for Quantum Science and Engineering (SIQSE), and Department of Physics, Southern University of Science and Technology (SUSTech), Shenzhen 518055, P.R.China.

^‡^These authors contributed equally to this work.

E-mails: [p-gao@pku.edu.cn](mailto:pengg@pku.edu.cn); [miwenbo@tju.edu.cn](mailto:miwenbo@tju.edu.cn); [xdbai@iphy.ac.cn](mailto:xdbai@iphy.ac.cn)

**S1. The magnetoresistance curve of SRO GB with and without grain boundary**

Fig. S1: The transport measurement of epitaxial single crystal SRO film deposited on STO single crystal(black) and SRO film with grain boundary on STO bicrystal (red).

**S2. EELS results at the grain boundary**


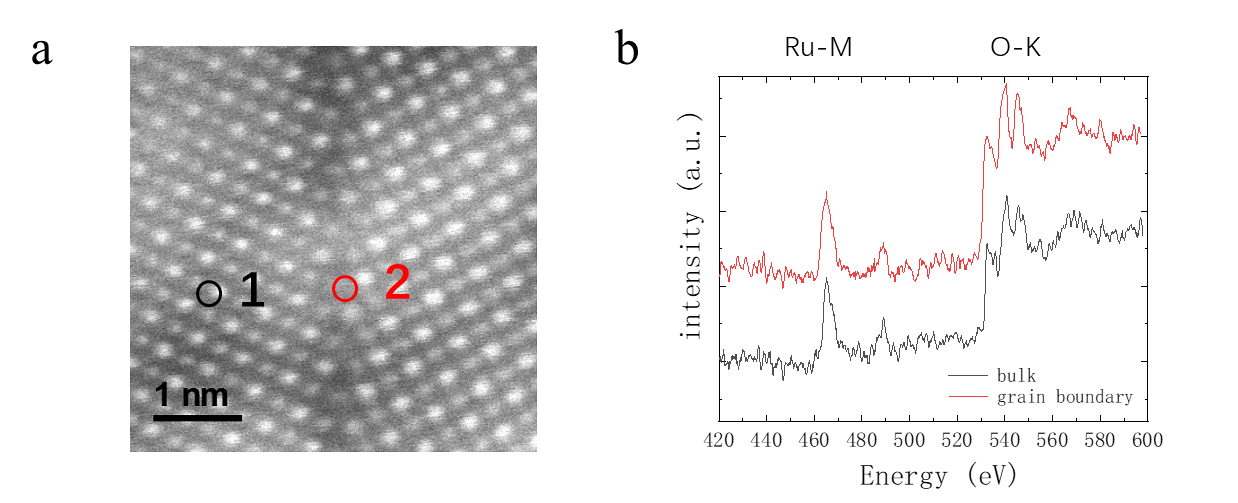


Fig. S2. EELS measurement of grain boundary. (a) the locations for EELS measurement. (b) the EELS were recorded at the bulk region (#1, black) and at the boundary (#2, red), respectively, indicating slight O accumulation at the grain boundary.

**S3. Simulation of symmetric and asymmetric structure**

In Table S1, a is the model obtained in our experiment, b is the model of symmetric O ions but asymmetric cations on both sides of grain boundary, and c is the model of symmetric O ions and cations on both sides of grain boundary, which is built based on the atomic structure of the same type of SrTiO_3_ grain boundaries reported in literature^1^. The Table S1 suggests that the asymmetric model ‘a’ has the lowest free energy and is more stable than the other two models. The Table S2 presents different structures in Table S1 exhibit different magnetic moment, indicating both of the structure and nonstoichiometry have significant influence on the magnetic properties.


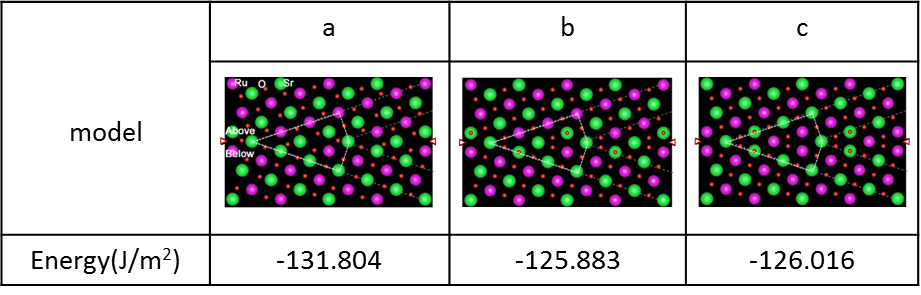


**Table** S1. DFT calculations of free energy for grain boundaries with different structures. Structures for (a) asymmetric, (b) symmetric O but asymmetric cations, (c) symmetric O and symmetric cations of SRO grain boundaries and the corresponding free energy.

| **Model** | | **a** | **b** | **c** | **Bulk** |
| --- | --- | --- | --- | --- | --- |
| Energy (J/m^2^) | | -131.804 | -125.883 | -126.016 | — |
| Sr:Ru:O ratio | | 14:12:38 | 14:12:38 | 13:13:38 | 1:1:3 |
| M_Ru_ (μ_B_) | Above | 0.134 | 0.046 | 0.941 | 1.525 |
| M_Ru_ (μ_B_) | Below | 1.166 | 0.141 | 1.048 | — |

Table S2. The free energy in symmetric and asymmetric SrRuO_3_ grain boundary models, corresponding to Table. S1. The magnetic moment of interfacial Ru atoms in SrRuO_3_ grain boundary and bulk.

**S4. Band structure**

The band structure of SRO grain boundary is intensively distributed compared with the bulk, but both exhibiting conducting properties.


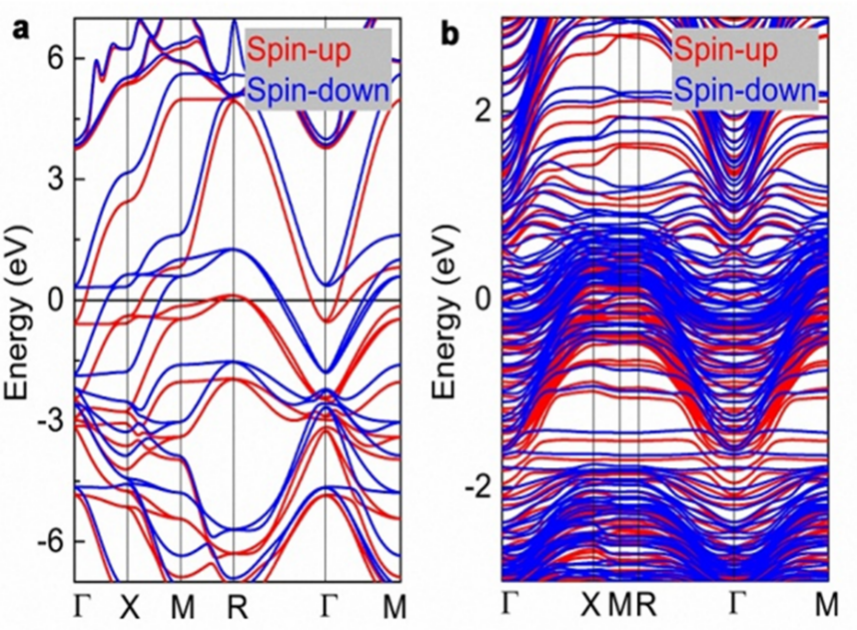


**Figure** S3. DFT calculations of band structure. Band structures of (a) SRO bulk and (b) grain boundary.

**S5. The magnetic moment**


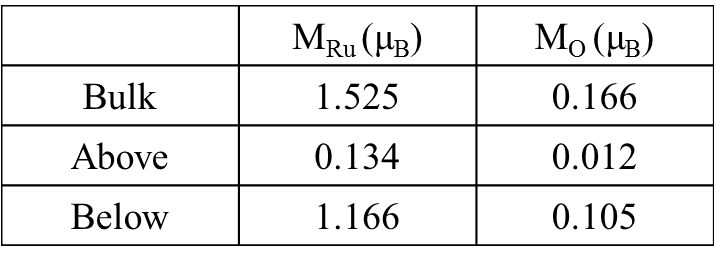


**Table** S3. DFT calculations of magnetic moments. Averaged magnetic moment (μ_B_) of Ru and O atoms in SRO bulk and grain boundary.

**S4. Length of** **O octahedron edges**

**
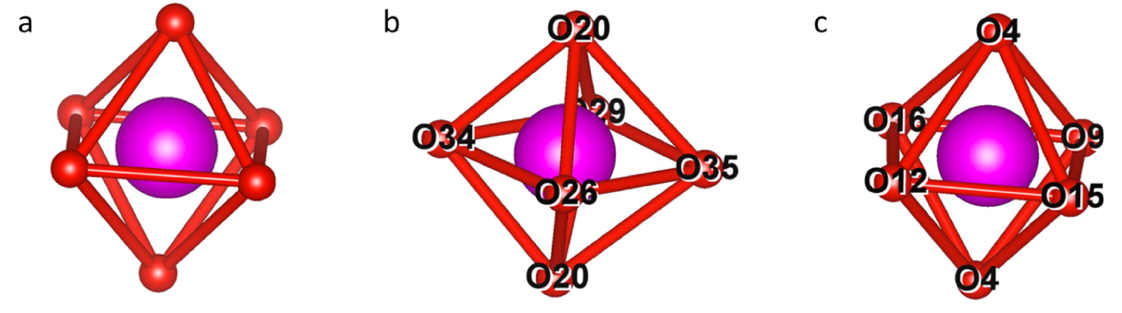
**

**Figure** S4. DFT calculations of RuO_6_ octahedron near the grain boundary. The RuO_6_ octahedral configurations of (a) bulk, (b) Ru-6, and (c) Ru-1.


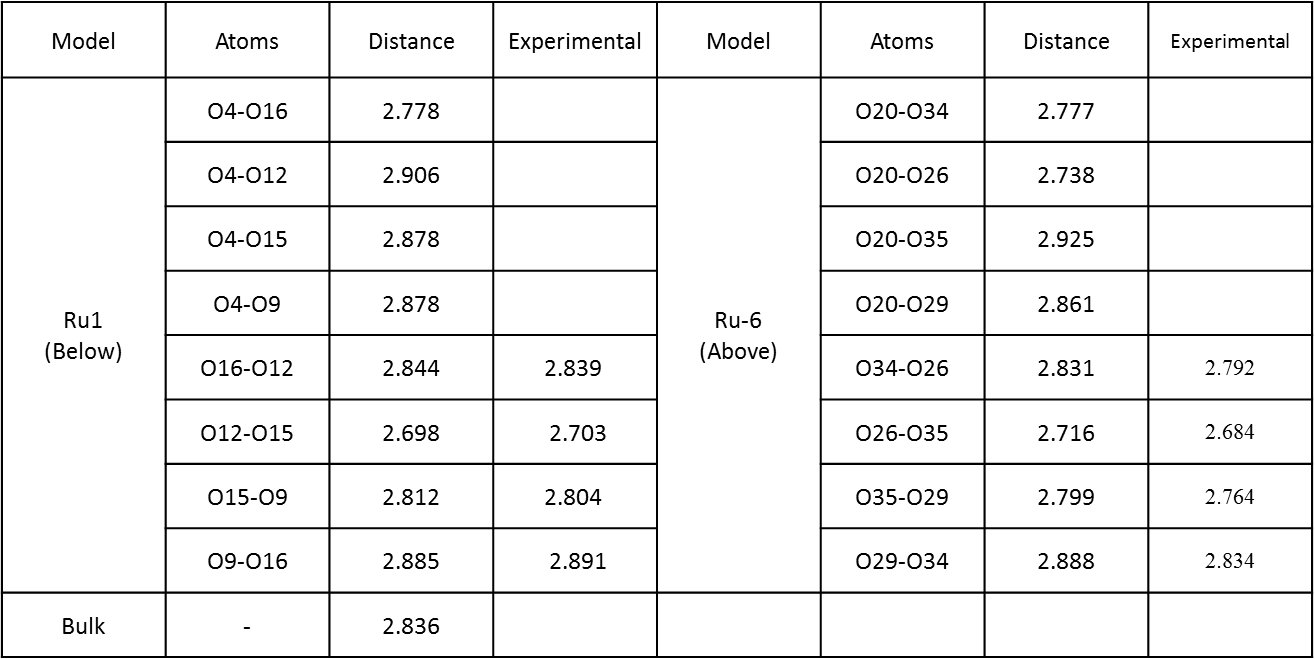


**Table** S4. The calculated atom distances. The distances (Å) of O octahedron for Ru1 and Ru 6 compared with the bulk.

**Supplementary references**

1 Ravikumar, V. & Dravid, V.P. Atomic structure of undoped Σ = 5 symmetrical tilt grain boundary in strontium titanate. *Ultramicroscopy* **52**, 557-563 (1993).
